# Spatiotemporal patterns of acute paracoccidioidomycosis hospitalizations in Brazil, 2014-2023

**DOI:** 10.1590/S1678-9946202567081

**Published:** 2025-11-10

**Authors:** Marina Cristina Gadêlha, Gustavo Cezar Wagner Leandro, Deisiany Gomes Ferreira, Amanda de Carvalho Dutra, Rosane Christine Hahn, Luciano de Andrade, Melyssa Negri

**Affiliations:** 1 Universidade Estadual de Maringá, Centro de Ciências da Saúde, Maringá, Paraná, Brazil; 2 Universidade Federal de Mato Grosso, Faculdade de Ciências Médicas, Cuiabá, Mato Grosso, Brazil

**Keywords:** Paracoccidioidomycosis, Hospitalization, Environment and public health, Spatio-temporal analysis

## Abstract

Paracoccidioidomycosis (PCM) is a neglected fungal disease with a rising incidence in Brazil, including increasing hospitalizations in previously non-endemic regions outside the Amazon. This study analyzed the spatiotemporal patterns of acute PCM hospitalizations and their relationship with deforestation. We carried out a retrospective ecological analysis of all PCM-related hospitalizations that were registered in the Brazilian Hospital Information System from 2014 to 2023. Acute and subacute cases were defined using ICD-10 codes (B40.0, B40.7, B40.8, B40.9, B41.0, B41.7, B41.8, and B41.9), whereas chronic forms were excluded. Rates were calculated with census population data and stabilized by Spatial Empirical Bayes smoothing. Space-time cube analysis was applied to detect clusters, which were then compared with deforestation alerts from the DETER-B satellite monitoring system. This study found 4,232 acute PCM hospitalizations, predominantly in men (80%), with a median age of 49 years. Cases were reported in 1,292 municipalities (23%), of which 78% were outside the Amazon. This study also found four significant hotspot clusters, including a newly emergent area in the Cerrado region along the Tocantins–Goias border near Brasilia. Hotspot municipalities showed the largest deforested areas (1,178 km^2^) when compared to coldspots (24 km^2^), suggesting a strong spatial association. The spatiotemporal dynamics of acute PCM indicate expansion into deforested areas of the Cerrado, highlighting the role of environmental disruption in shaping disease risk. Mitigating PCM spread in Brazil urgently requires strengthened surveillance and integrated health–environmental policies.

## INTRODUCTION

Paracoccidioidomycosis (PCM) is a neglected tropical fungal infection caused by *Paracoccidioides spp.*, dimorphic fungi that predominantly affect men with a history of soil-related occupations, such as agriculture, forestry, and other land-disturbing activities. The disease often presents itself in its chronic form, leading to significant pulmonary impairment if left untreated^1^. Brazil accounts for 80% of cases of this endemic mycosis in Latin America, particularly in regions near Amazon deforestation^2^. Research suggests that infections and the disease caused by *Paracoccidioides spp*. are linked to environmental factors in agricultural areas, such as sandy, acidic soils, disturbed vegetation, and proximity to water bodies, which may influence the distribution of the pathogen and risk of exposure in individuals^3^.

While paracoccidioidomycosis is one of the main endemic mycoses in Brazil, it is crucial to differentiate it from blastomycosis, another fungal infection^4^. Blastomycosis is caused by the fungus *Blastomyces dermatitidis* and is endemic to North American soils, such as the Ohio and Mississippi River Valleys^5^. Notably, Brazil has no documented cases of North American blastomycosis^5^. However, the confusion between the two diseases is historical. Until the early 1970s, the Brazilian infection was called South American blastomycosis, and the American one, North American blastomycosis^3,5^. To correct this imprecision and acknowledge the widespread distribution of *Paracoccidioides* in Central and South America, South American blastomycosis was renamed paracoccidioidomycosis in 1972^4^. Despite this change, the generic and isolated use of the term "blastomycosis" remains common in the medical field, which contributes to the confusion.

The World Health Organization has designated PCM as a priority pathogen, emphasizing its significant health burden and calling for enhanced global efforts to improve surveillance, prevention, treatment, and the development of new diagnostic tools and therapies^6^. National epidemiological information remains scarce in Brazil due to underreporting, limited monitoring, and heterogeneous healthcare infrastructure. In 2020, the Brazilian list of compulsory notifiable diseases incorporated PCM (Portaria Nº 264/2020) as an attempt to improve awareness and epidemiological monitoring. However, the most recent regulation (Portaria GM/MS Nº 5.201/2024) no longer includes PCM in this list, underscoring the instability of national surveillance policies for this mycosis^7,8^.

While chronic PCM is the more common presentation, the disease also manifests itself in a non-chronic or acute/subacute form known as juvenile PCM. This form affects 5%–10% of cases in children and adolescents in endemic areas, although it can also occur in adult men. Non-chronic PCM may also develop opportunistically in immunosuppressed patients^3^. The non-chronic form of the fungus can be disseminated to the lymphatic and hematogenous systems, spreading across organs and systems and leading to more disseminated lesions and clinical manifestations that appear rapidly (within weeks or months after the infection)^3,9^. These cases often show a severe and aggressive clinical picture. Thus, studies on non-chronic cases can serve as sentinel events to monitor the disease in endemic regions^2,9^.

Recent studies have shown a concerning increase in hospitalizations and mortality from acute PCM outside the Amazon, particularly in metropolitan areas such as Rio de Janeiro. This trend suggests a rise in more severe cases, younger patients, and longer hospital stays^10,11^. The spread of the fungus into urban areas, coupled with the increase in acute cases, underscores its potential adaptability to different environments and highlights significant gaps in current surveillance and mitigation strategies. To better understand this issue, we investigated the spatiotemporal patterns of acute PCM hospitalizations in Brazil from 2014 to 2023.

## MATERIALS AND METHODS

A retrospective ecological analysis was conducted on the 2014–2023 period. Hospitalization data for PCM were obtained from anonymized, publicly available records of the Hospital Information System of the Brazilian Unified Health System. Data were accessed in June 2024 using the microdatasus package on R (version 4.4.1, R Foundation for Statistical Computing, Vienna, Austria) via the National Information System transfer interface^12^.

The dataset encompassed non-chronic PCM admissions from 2014 and 2023, with primary diagnoses classified under the subcategories B40 and B41 of the International Classification of Diseases (ICD). The ICDs of blastomycosis (B40) were also included since, in Brazil, this disease (South American blastomycosis) is considered the same as paracoccidioidomycosis^13,14^. Only the admissions related to non-chronic cases of PCM were included in this study. Thus, only the records the following categories as their main diagnosis were maintained: acute pulmonary blastomycosis (B40.0), disseminated blastomycosis (B40.7), other forms of blastomycosis (B40.8), unspecified blastomycosis (B40.9), pulmonary PCM (B41.0), disseminated PCM (B41.7), other forms of PCM (B41.8), unspecified PCM (B41.9). The categories chronic pulmonary blastomycosis (B40.1), chronic pulmonary blastomycosis unspecified (B40.2), and blastomycosis cutanea (B40.3) were excluded.

To estimate smoothed hospitalization rates per 100,000 residents, population census data and applied Spatial Empirical Bayes smoothing technique were used, reducing the impact of underreporting and accounts for the inherent data variability^15^.

Spatiotemporal clusters of PCM cases were identified by a space-time cube analysis on ArcGIS (version 3.1, Esri, Redlands, CA, USA). Such analysis operates on a three-dimensional spatiotemporal framework that was generated by aggregating point data, in which each bin represents a spatial location and temporal interval. Using the spatial and temporal neighborhood, the tool applies the Getis-Ord Gi* statistic to calculate z-scores, p-values, and hot or coldspot classifications for each bin. Subsequently, the temporal sequence of hot and coldspot designations within each location is assessed using the Mann-Kendall trend test, which provides additional z-scores and p-values to evaluate monotonic trends. Based on these statistical outputs, each location is categorized into a designation (e.g., new, consecutive, intensifying, persistent, diminishing, sporadic, oscillating, historical hot or coldspots), thereby enabling a robust spatiotemporal classification of clustering patterns^16^.

Deforestation data were obtained from the DETER-B (real-time deforestation detection) system, which uses satellite imagery to track near real-time forest cover changes. The system provides daily alerts that monitor deforestation across critical biomes, including the Amazon, Cerrado (tropical savanna), and Pantanal, offering valuable insights into environmental changes that may influence PCM spread^17^.

As this research with aggregated information had no possibility of individual identification, this study was exempted from registration and ethical evaluation via the CEP/CONEP system (Official Letter Nº 05/2020) in accordance with current regulations.

## RESULTS

**Figure 1:**
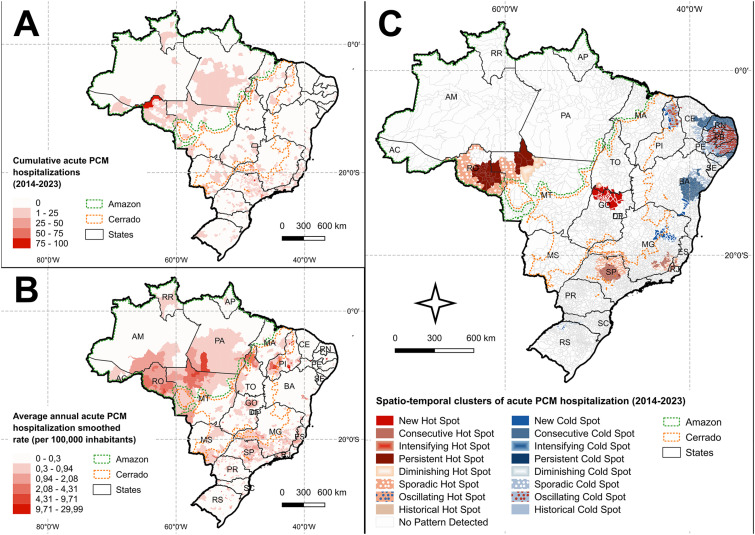
Spatiotemporal patterns of acute paracoccidioidomycosis hospitalizations in Brazil, 2014-2023: (A) cumulative acute PCM hospitalizations; (B) average annual acute PCM hospitalization smoothed rate (per 100,000 inhabitants); (C) spatiotemporal clusters identified across municipalities.

Brazil recorded 4,232 hospital admissions for acute PCM from 2014 to 2023. Of these cases, 80% occurred in men with a median age of 49 years—although the age range spanned from one to 98 years, indicating that the disease affects a broad age spectrum. Among the hospitalizations, 22% (n=917) were reported in the Amazon region, whereas the remaining 78% (n=3,315) occurred in other areas. PCM hospitalizations were recorded in 1,292 out of the 5,570 Brazilian municipalities (23%), highlighting the widespread geographic distribution of the disease across all macro-regions of the country (Figures 1A and 1B).

The spatiotemporal analysis found four significant hotspots of acute PCM hospitalizations. The first hotspot, a newly identified area in 2022 is in the Cerrado region along the border between Tocantins and Goias State encompassed 25 municipalities. The second region, spanning Rondonia and Mato Grosso, has historically high but intermittent hospitalization rates, with borders marked by areas experiencing declining rates and others with occasional peaks. The third zone, between Sao Paulo and Minas Gerais, includes five central municipalities with consistently high but intermittent hospitalization rates. These municipalities are surrounded by areas with sustained high rates in recent years, further encircled by regions marked by sporadic hospitalization peaks. The fourth area, covering Minas Gerais, Rio de Janeiro, and Espirito Santo, has consistently high hospitalization rates, with neighboring regions showing irregular fluctuations in hospitalization (Figure 1C).

In contrast, the spatiotemporal analysis also found four distinct coldspot clusters. The largest coldspot lies in the Northeast, primarily covering Paraiba, Pernambuco, and Rio Grande do Norte, with smaller clusters in Alagoas and Ceara. This cluster features a central area that has experienced high hospitalization rates but has seen a significant decline in recent years, surrounded by regions with no reported hospitalizations in the most recent periods. A similar pattern occurs in another coldspot cluster in northern Piaui up to its border with Ceara. The coldspot cluster in Bahia mainly consisted of municipalities with low hospitalization rates in recent years, with a smaller portion including areas with sporadically low rates and newly found coldspots. A new coldspot cluster also occurred in the Southeast, where acute PCM hospitalization rates have significantly decreased for the first time in the most recent analysis period. Additionally, a sporadic coldspot cluster emerged in northern Rio Grande do Sul, indicating that the area has consistently showed below average acute PCM hospitalizations rates during the analyzed period, although intermittently and inconsistently (Figure 1C).

**Figure 2:**
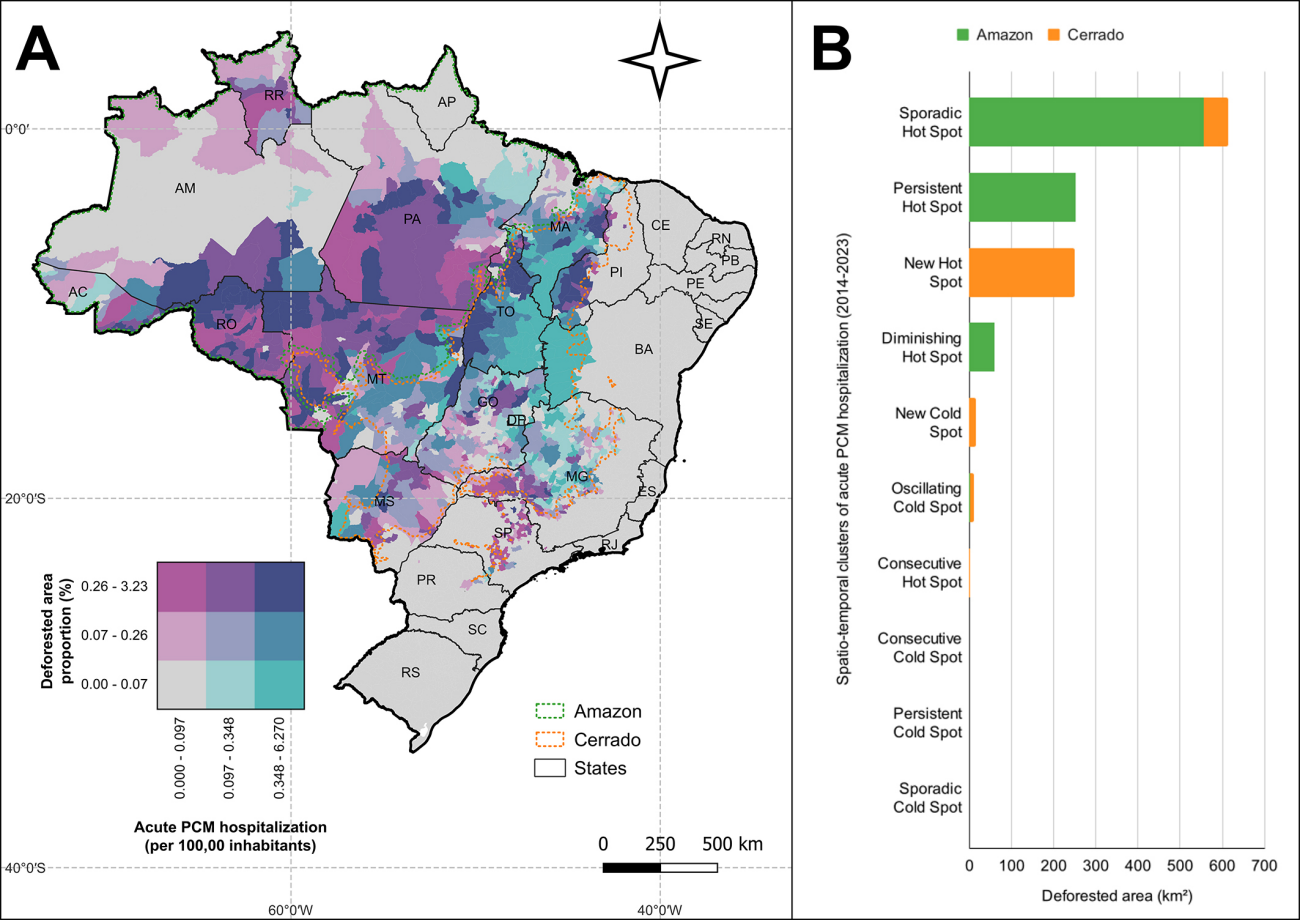
Spatial association of deforestation area with acute PCM hospitalization in the Amazon and Cerrado biomes of Brazil, 2014-2023: (A) bivariate analysis of deforested area proportion (%) and acute PCM hospitalization (per 100,000 inhabitants); (B) deforested area (km²) by spatiotemporal clusters in the Amazon and Cerrado biomes.

In the Amazon region, deforestation and acute PCM hospitalizations are notably high in areas of Rondonia, Para, and Mato Grosso, indicating a strong correlation between environmental degradation and the spread of the disease in these highly affected regions. A similar geographical trend occurred in the Cerrado region, particularly in Maranhao, Piaui, and Tocantins State, which showed significant deforestation and increased hospitalizations rates. Smaller areas within Goias, Minas Gerais, and Mato Grosso do Sul also showed modest levels of deforestation and higher hospitalization rates, although these trends were less pronounced. In the Cerrado, deforestation and hospitalizations are especially prevalent in border areas, suggesting that the disease is spreading inward in regions with significant deforestation (Figure 2A).

A comparative analysis of deforested areas across different spatiotemporal cluster types showed that municipalities with higher hospitalization rates for acute PCM had the largest deforested areas. Municipalities classified as hotspots, regardless of cluster type, consistently showed more extensive deforestation (1,178.143 km^2^) than coldspots (24.004 km²). This pattern suggests a correlation between deforestation and higher hospitalization rates for acute PCM as hotspots experience more severe environmental degradation. Notably, municipalities classified as sporadic hotspots, which show elevated but inconsistent hospitalization rates, had the highest rates of deforestation, with the Amazon region being particularly affected. Furthermore, a recurring trend of new hotspots emerging concentrated itself in the Cerrado. This shift in disease distribution indicates that the region is experiencing increasing levels of deforestation and acute PCM hospitalizations (Figure 2B).

## DISCUSSION

Outbreaks of PCM have been linked to deforestation and extensive land disturbance during highway construction in Rio de Janeiro, suggesting that major environmental disruptions, such as large-scale land use changes, may facilitate the transmission of the fungus^18^. Recent studies highlight that northern Tocantins, now reclassified as hyperendemic, faces a significantly higher risk of PCM infection due to ongoing deforestation, agricultural expansion, and the encroachment of human settlements^19^. This underscores the urgent need for updated health surveillance criteria to address the impacts of urbanization, population migration, and increasing environmental degradation^19^.

Our study found a novel hotspot for acute PCM in 25 municipalities along the border between Tocantins and Goias, particularly near the federal capital, Brasília. The rapid rate of deforestation and ongoing agricultural expansion, which are substantially altering the local ecosystem^3^, have exacerbated this emerging hotspot. The findings indicate that regions undergoing intense deforestation are more likely to emerge as new hotspots for PCM as the disruption of natural ecosystems likely increases human exposure to *Paracoccidioides*. This underscores the necessity for targeted monitoring and intervention in these at-risk areas, particularly in the Brazilian North, in which 30% of patients are compelled to seek treatment in other areas. This highlights significant challenges in healthcare access for rural populations. Plus, the mobility of infected individuals emphasizes the need to recognize urban centers near deforested areas as potential focal points for disease transmission given the heightened human-environment interactions in these areas^1,2^.

Moreover, our findings highlight significant deficiencies in diagnosing and treating PCM within the Brazilian healthcare system. A key challenge refers to the reliability of hospital records, which suffers from diagnostic and coding errors that often misclassify paracoccidioidomycosis (B41) as blastomycosis (B40)^13,14^. We faced a significant methodological bias because, despite the lack of documented blastomycosis cases in Brazil, we had to consider ICD records for blastomycosis (B40) in our epidemiological data analysis due to the persistent historical confusion in terminology between the two diseases^3,4^. We believe that these blastomycosis records in Brazil actually correspond to misclassified cases of paracoccidioidomycosis. Ignoring this data would substantially underestimate the true incidence of the disease, making their inclusion essential for a more complete evaluation.

A limitation of this study refers to its reliance on hospitalization records, which exclude milder cases of PCM, potentially leading to an underestimation of the true disease burden. To mitigate this limitation, we applied the spatial empirical Bayes smoothing, leveraging local spatial information to refine rate estimates. This approach helps to reduce random fluctuations, particularly in areas with small populations, enhancing the stability of the estimates and accounting for underreporting and data sparsity. Another limitation pertains to potential biases in deforestation data derived from satellite imagery as cloud cover may obstruct the detection of deforestation events. Despite this challenge, this study represents the first effort to incorporate satellite-based deforestation data into spatial analyses of acute PCM, underscoring its significance in understanding the environmental factors that influence disease dynamics.

## CONCLUSION

In conclusion, the spatiotemporal dynamics of acute PCM hospitalizations in Brazil show important epidemiological trends, notably the rising number of cases outside the Amazon, particularly in newly identified high-risk areas within the tropical savanna biome. This study underscores the critical need for integrated surveillance and response systems that can monitor PCM cases and incorporate deforestation data. Such an approach is vital to effectively manage the spread of PCM, particularly in regions under rapid environmental changes, to mitigate the impact of the disease and prevent further outbreaks across Brazil.

## Data Availability

The complete anonymized dataset supporting the findings of this study is available from: https://osf.io/8mb2g
